# Frailty; Time for Global Action: Commentary on “Frailty and its Association with Long-Term Mortality among Community-Dwelling older adults aged 75 years and Over”

**DOI:** 10.1186/s13584-024-00646-4

**Published:** 2024-10-04

**Authors:** Yotam Weiss, Idit Matot

**Affiliations:** Division of Anesthesia, Intensive Care, and Pain Management, Tel-Aviv Medical Center, Tel- Aviv University, 6 Weizmann St, Tel-Aviv, 6423906 Israel

**Keywords:** Frailty, Frailty Index, Long-term mortality, Older adults, Israel, Clinical practice, Health policy

## Abstract

This commentary examines the study “Frailty and Its Association with Long-Term Mortality Among Community-Dwelling Older Adults Aged 75 Years and Over” by Lewis et al. The retrospective cohort study utilized data from a primary healthcare provider in Israel to investigate frailty using the Frailty Index (FI) and its correlation with long-term mortality. Nearly half of the older adult cohort was identified as frail, with a strong association between higher frailty levels and increased mortality risk. The commentary emphasizes the importance of routine frailty screening in clinical practice and health policy. Integrating FI calculations into electronic health records can facilitate timely care for high-risk individuals. However, presenting frailty data must be managed carefully and in conjunction with patients’ preferences to avoid stigmatizing and negatively influencing clinical decisions. While the FI is a valuable tool, it should complement, not replace, other assessments that provide a more holistic view of the patient’s health. Furthermore, the commentary strongly advocates for a more comprehensive approach to patient care, emphasizing that non-geriatricians must also be proficient in recognizing and managing frailty. Effectively addressing frailty can lead to significant cost savings for healthcare systems, reduced burden on healthcare facilities, and decreased need for long-term care.

## Main text

The study “Frailty and Its Association with Long-Term Mortality Among Community-Dwelling Older Adults Aged 75 Years and Over” by Lewis et al. presents a comprehensive analysis of frailty prevalence and its impact on mortality among older adults in Israel. This large-scale, retrospective cohort study utilized data from the third-largest healthcare provider in Israel to investigate frailty using the cumulative deficit method (the Frailty Index [FI]) and its correlation with long-term mortality in a nationwide unselected population.

The study’s primary finding is the high prevalence of frailty among Israeli older adults, with nearly half of the cohort identified as frail. Additionally, the study highlights the well-established association between frailty and increased mortality, revealing a clear trend where higher levels of frailty correlate with significantly elevated mortality risk.

Consistent with global findings, the prevalence of frailty in older adults ranges from 4 to 60%. This broad range is likely due to different definitions and methodologies for assessing frailty [1, 2]. The FI, based on the cumulative deficit method, considers a comprehensive range of age-related health deficits and chronic diseases. The use of the FI in this study aligns with other research, suggesting that this method provides a more accurate prediction of adverse outcomes, including mortality, compared to age alone [3, 4]. However, the primary challenge of the FI is that it requires at least 30 health variables to be accurately coded within health records [4, 5]. Other tools, such as the frailty phenotype, focus on specific clinical criteria like weight loss, exhaustion, physical activity, grip strength, and walking speed [6, 7]. Another widely used tool is the Clinical Frailty Scale (CFS), which is a subjective measure based on clinical judgment and functional status [8]. A recent meta-analysis concluded that the FI and CFS are shorter and easier to apply, and the frailty phenotype requires more specific physical assessments and tools. All tests accurately predict unfavorable outcomes, but their applicability may vary depending on the clinical context and the patient population [9, 10]. 

The study identified a higher prevalence of frailty among females, non-Orthodox Jews, and individuals with multiple chronic conditions. The higher prevalence among females, despite their lower mortality rates, underscores the complex interplay between gender, frailty, and survival. Biological, social, and behavioral factors likely contribute to this phenomenon. Women generally live longer than men, leading to a higher proportion of women in older age groups where frailty is more common. Additionally, women may be more likely to report health deficits and seek medical attention, resulting in higher recorded frailty scores. Despite being more frail, women often have better survival rates than men, possibly due to differences in the types of chronic conditions they experience, their health behaviors, and their biological resilience [11, 12].

The data clearly show an association between frailty and increased mortality, with a clear gradient observed across mild, moderate, and severe frailty levels. Mortality rates were significantly higher among frail individuals, with the hazard ratios indicating a strong independent association between frailty severity and mortality, even after adjusting for age, gender, and population sector [13].

The study’s findings have significant implications for clinical practice and health policy. Routine frailty screening can help identify high-risk individuals and implement targeted interventions to potentially improve their health outcomes [14, 15]. In an electronic medical record system where general practitioners code the patients’ medical conditions and medications, the FI should be automatically calculated and integrated into health records, similar to other vital signs and measures of functional capacity. Making frailty a routine part of patient assessments can facilitate timely and appropriate care [5]. The recent review by Kim et al. reinforces frailty’s role as a critical predictor of adverse outcomes and highlights the importance of routine screening in high-risk settings like surgery and oncology, where interventions such as exercise, nutritional support, and comprehensive geriatric assessments show promise but face challenges in consistent implementation.​ [7].

However, as the authors addressed, presenting frailty data in the medical chart might stigmatize older adults and even deprive them of a suggested therapy (automatically assuming they are not fit for surgery, for example). Labeling patients as severely frail might lead to negative biases and lower expectations for recovery or rehabilitation, thereby influencing clinical decisions [16]. Additionally, the absence of consideration for quality of life or outcomes other than death highlights potential limitations in relying solely on the FI for clinical decision-making. Quality of life, patient preferences (“what matters”), and functional outcomes are critical factors that should complement frailty assessments to ensure holistic and patient-centered care [17, 18]. Therefore, while the FI is a valuable tool that should be automated, it should not replace other assessments to provide a comprehensive understanding of a patient’s health status and guide appropriate interventions.

Frailty screening by non-geriatricians is of utmost importance. Given the high prevalence of frailty and its impact on mortality, all healthcare providers need to be adept at recognizing and managing frailty. In any setting, frail and at-risk patients might benefit from tailored interventions such as nutritional support, physical therapy, and management of chronic conditions [19, 20]. This includes the perioperative setting. As the population ages, older adults are undergoing more surgeries than ever before [21]. Preoperative frailty assessment is crucial for anesthesiologists to evaluate the risk of surgery in older adults. Frail patients are at higher risk for postoperative complications, including delirium, prolonged hospital stays, and increased mortality. Incorporating frailty screening into the preoperative evaluation can guide anesthesiologists in developing tailored anesthesia plans, optimizing predisposing factors, and making informed decisions about the suitability of surgical interventions [9, 22].

Beyond screening, identifying frail patients as a health policy initiative holds significant potential for prehabilitation and rehabilitation programs. Prehabilitation, which includes exercise and cognitive training, nutritional optimization, and psychological support before surgery, is designed to enhance patients’ resilience. This preparation can reduce postoperative complications by strengthening patients physically and mentally before the stress of surgery [23, 24]. Rehabilitation (or post-habilitation) focuses on the recovery phase, aiming to restore functional status through continued physical therapy, nutritional support, and chronic condition management. This approach aims to improve long-term outcomes and reduce the likelihood of readmissions [25]. Integrating pre- and rehabilitation strategies into perioperative care provides a comprehensive and holistic approach to managing frail surgical patients, ensuring better overall health and recovery [26].

Despite its strengths, the study has limitations, including reliance on formal diagnoses documented for clinical purposes, which may introduce information biases [27]. Additionally, the study’s findings are based on a specific population within Israel, and the overrepresentation of certain demographic groups may limit the generalizability of the results. Future research should validate these findings in different populations, investigate the integration of automated FI assessments into clinical practice, and explore the clinical and financial impact of interventions designed to manage frailty [14].

## Conclusions

To conclude, the study by Lewis et al. provides compelling evidence of the high prevalence and significant impact of frailty on long-term mortality among older adults in Israel. The study’s findings have significant implications for clinical practice and health policy. Assimilating frailty assessments into routine care can enhance decision-making and care planning at the clinical level. Routine frailty screening over time can help identify high-risk individuals and recommend early targeted interventions to prevent frailty progression, which might improve their quality and quantity of life. The financial benefits of preventing frailty progression are substantial. Fewer falls, fractures, and hospitalizations translate to significant cost savings for healthcare systems, reduced burden on healthcare facilities, and decreased need for long-term care [28]. For policymakers, the study underscores the need for systems-level changes to incorporate frailty screening into electronic health records and support resource allocation for frailty management programs [29]. As the population ages, the importance of addressing frailty in clinical practice and health policy cannot be overstated. This study serves as a critical reminder of the need for proactive measures to manage frailty and improve the health and well-being of older adults.

## Data Availability

Not relevant.

## Abbreviations

FI: Frailty Index
CFS: Clinical Frailty Scale
